# Aberrant Gut-To-Brain Signaling in Irritable Bowel Syndrome - The Role of Bile Acids

**DOI:** 10.3389/fendo.2021.745190

**Published:** 2021-11-30

**Authors:** Róisín Ní Dhonnabháín, Qiao Xiao, Dervla O’Malley

**Affiliations:** ^1^ Department of Physiology, College of Medicine and Health, University College Cork, Cork, Ireland; ^2^ APC Microbiome Ireland, University College Cork, Cork, Ireland

**Keywords:** TGR5, FXR, lithocholic acid, microbiome, IBS, bile salt hydrolase

## Abstract

Functional bowel disorders such as irritable bowel syndrome (IBS) are common, multifactorial and have a major impact on the quality of life of individuals diagnosed with the condition. Heterogeneity in symptom manifestation, which includes changes in bowel habit and visceral pain sensitivity, are an indication of the complexity of the underlying pathophysiology. It is accepted that dysfunctional gut-brain communication, which incorporates efferent and afferent branches of the peripheral nervous system, circulating endocrine hormones and local paracrine and neurocrine factors, such as host and microbially-derived signaling molecules, underpins symptom manifestation. This review will focus on the potential role of hepatic bile acids in modulating gut-to-brain signaling in IBS patients. Bile acids are amphipathic molecules synthesized in the liver, which facilitate digestion and absorption of dietary lipids. They are also important bioactive signaling molecules however, binding to bile acid receptors which are expressed on many different cell types. Bile acids have potent anti-microbial actions and thereby shape intestinal bacterial profiles. In turn, bacteria with bile salt hydrolase activity initiate the critical first step in transforming primary bile acids into secondary bile acids. Individuals with IBS are reported to have altered microbial profiles and modified bile acid pools. We have assessed the evidence to support a role for bile acids in the pathophysiology underlying the manifestation of IBS symptoms.

## Introduction

Over a hundred trillion microbial organisms, mostly bacteria, inhabit the human colon and have co-evolved with their hosts to have diverse, but primarily beneficial, functions. They scavenge additional calories by fermenting non-digestible foods, secrete vitamins and ensure normal physiological development. A plethora of studies have demonstrated that microbes have the capacity to modulate host physiological homeostasis and have been linked with cognitive disorders such as anxiety, depression, Parkinson’s disease, autism spectrum disorder and schizophrenia (1, 2), in addition to the development of inflammatory bowel disease (3) and irritable bowel syndrome (IBS) (4). Several direct and indirect mechanisms of cross-barrier communication have been proposed, where microbial, endocrine or immune factors are posited as inter-kingdom signaling molecules (5–7). In this review however, we will focus on bile acids, liver-derived bioactive host molecules that exhibit an interdependency with resident intestinal bacteria.

**Figure 1:**
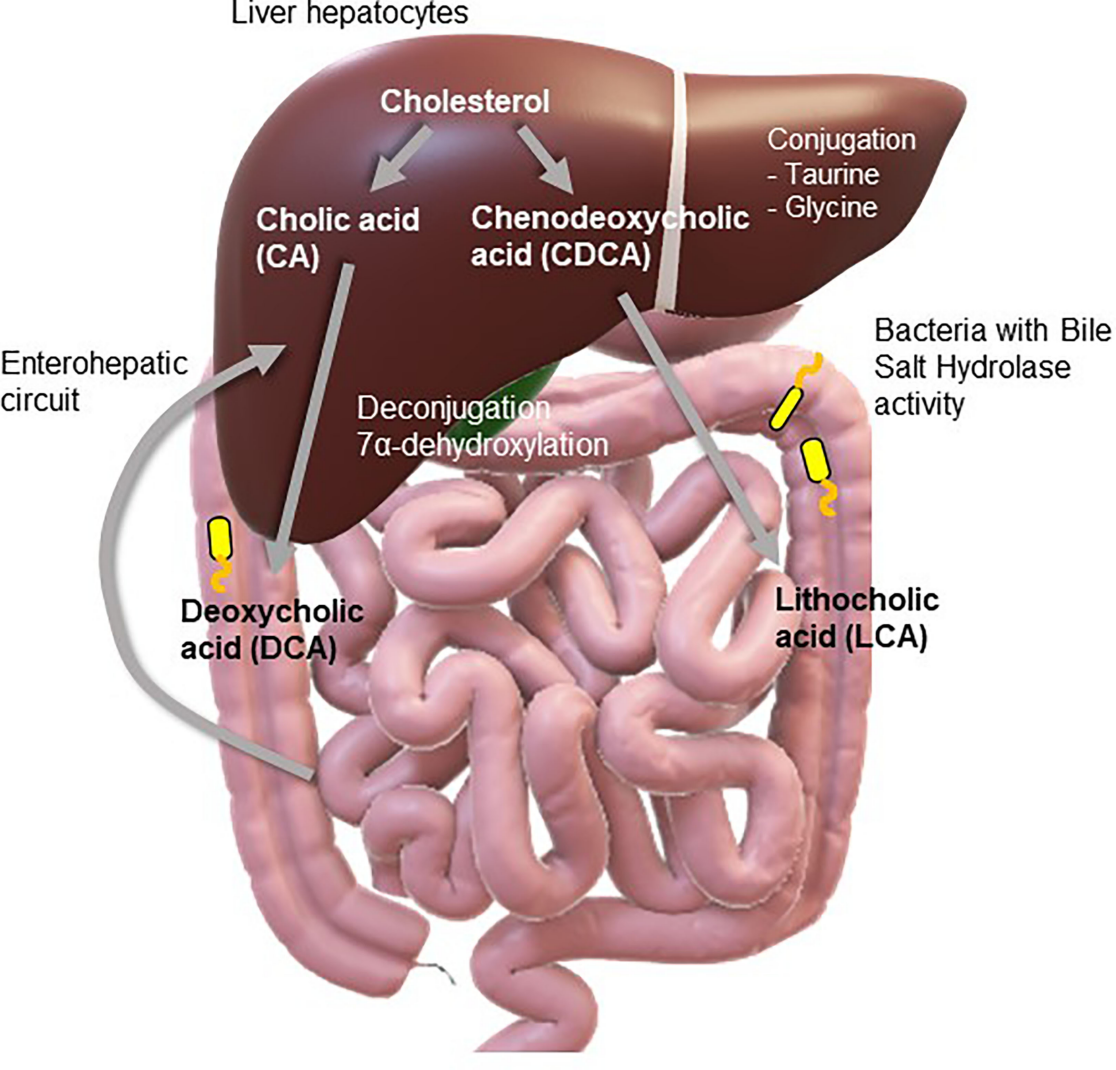
Bile acid synthesis. A dynamic relationship exists between microbes and bile acids with both modifying the profiles of the other. Liver hepatocytes synthesize primary bile acids (cholic acid and chenodeoxycholic acid) from cholesterol. It may then be conjugated within the hepatocytes with taurine or glycine. After bile flows into the intestine, it encounters bile salt hydrolaze (BSH)-containing bacteria, which transform cholic acid and chenodeoxycholic acid into secondary bile acids such as deoxycholic acid (DCA) and lithocholic acid (LCA). A plethora of secondary bile acids are produced through deconjugation of the amino acids, glycine or taurine, and dehydroxylation, dehydrogenation, and epimerization of the cholesterol core. Secondary bile acids returned to the liver by the enterohepatic circuit can also be conjugated to taurine or glycine. Moreover, amino acids may also be conjugated to bile acids further increasing the diversity of human bile acids.

Hepatocytes synthesize and secrete the primary bile acids, cholic acid (CA) and chenodeoxycholic acid (CDCA), into the duodenum *via* the biliary ductal system. Comprising about half of the total solutes in bile, their amphipathic structure facilitates emulsification and subsequent digestion and absorption of dietary lipids being emptied from the stomach (8). The enterohepatic circuit is an extremely efficient method whereby ~95% of bile acids are reabsorbed in the terminal ileum and returned *via* the portal vein to the liver, where they are taken up by hepatocytes and re-secreted into the bile ducts. Just 5% of bile acids escape reuptake and spill over into the colon, the intestinal site with the highest density of microbes. A dynamic, symbiotic relationship exists between microbes and bile acids (**Figure 1**), resulting in a great diversity of microbially-modified secondary bile acids (9). Bile salt hydrolaze (BSH)-containing bacteria hydrolyze and deconjugate taurine or glycine from the sterol core of the primary bile acids, facilitating further passive reabsorption in the colon. This process also enables further microbially-mediated transformations to produce a plethora of secondary bile acids, including deoxycholic acid (DCA) and lithocholic acid (LCA). This results in an enrichment of secondary bile acids in the colon, where their chemical characteristics help shape bacterial profiles within the microbiome (10). Given that many different cell types express bile acid receptors (11–14) and both active and passive transport of bile acids across the gut barrier and subsequent uptake into the portal vein distributes bile acids to extra-intestinal peripheral organs, bile acids are classified as bioactive signaling molecules (15). We have examined the potential role of bile acids to modify host physiological homeostasis, with a focus on gut-brain axis signaling and their potential role in IBS-related bowel dysfunction.

## Intestinal Profile of Bile Acids

Biliary secretions are comprised of bile salts, pigments, water, and waste products, including bilirubin and excess cholesterol. In humans, the primary bile acids, CA and CDCA are synthesized and conjugated with either glycine or taurine (16) and stored, concentrated and acidified in the gall bladder prior to being released into the duodenum, along with pancreatic enzyme secretions. Bile acids comprise a family of closely related acidic sterols with similar, but not identical chemical structures and detergent performance. Conjugation of bile acids alters their pKa meaning bile salts are almost always in a protonated form in the duodenum, thereby restricting their passive movement through the small intestinal epithelial barrier. Apical sodium-dependent bile salt (ABST) transporters in the distal ileum facilitate active transport across the gut barrier prior to their return to the liver *via* the portal vein. The ~ 5% of bile acids which reach the colon, are either reabsorbed *via* passive diffusion or lost in the feces (17).

Bile acid metabolism is influenced by bacteria with BSH activity, a property that is unique to gut-residing bacteria and may have evolved through host-driven selection (18). An incentive for bacterial participation in this interaction may be the acquisition of the glycine and taurine conjugates for their own metabolic needs, in addition to the disposal of excess electrons generated during fermentation processes (19). Moreover, tolerance of bile may confer an advantage to these microbes in terms of their ability to colonize gut regions (20). In addition to acting as a nutrient source for intestinal bacteria and providing environmental cues, bile acids are also noted for their antimicrobial properties either through direct cytotoxicity (21) or by stimulating innate immune mechanisms (22). This constrains small intestinal bacterial overgrowth (23). Diarrhea resulting from exposure to high levels of bile acids in the colon, may be part of the innate immune response to protect the intestinal epithelium from cytotoxic bile acids, such as LCA (24).

In a bi-directional arrangement, bacterial enzymes chemically modify bile acids, and in turn, bile acids modify gut bacterial profiles. Cleavage of amino acid side chains on glycine- or taurine-conjugated primary bile acids changes their physiochemical properties, such that they are more lipophilic and susceptible to further modification by bacteria, including 7α-dehydroxylation, dehydrogenation and epimerization (21, 25, 26). Secondary bile acids, such as DCA and LCA, may undergo further modification, including sulphation and glucuronidation, imparting changes in their lipophilicity and hydrophilicity. Moreover, the potency of secondary bile acids for bile acid receptors differs from primary bile acids (23) and the amphipathic nature of bile acids can directly affect the physical properties of cellular lipid membranes, thereby modifying cell signal transduction (27). This has consequences for local signaling and gut homeostasis (28).

## Molecular Mechanisms Underlying Bile Acid Signaling

Given the diversity of bile acids identified in mammals and the variety of bile acid receptors with variable binding affinities and response potencies, it is no surprise that the bioactive functions of bile acids differ significantly. Farnesoid X (FXR) is the most extensively studied nuclear bile acid receptor, for which CDCA is the most potent agonist (15), however there are also other bile acid-sensitive nuclear receptors, such as pregnane X (PXR) and vitamin D receptors (VDR), which are expressed on several different cells types (11–14, 29–31). To activate nuclear bile acid receptors, bile acids must cross cellular and nuclear lipid bilayers, a process that can occur by passive diffusion or be facilitated by active transport (15, 32, 33). FXR is translocated to the cell nucleus upon activation, where it forms a heterodimer with retinoid X receptor and binds to hormone response elements present on DNA (34), instigating changes in gene regulation. One of the primary functions of FXR activation by bile acids is the feedback inhibition of bile acid synthesis through the suppression of CYP7A1, the rate-limiting enzyme in the classical bile acid synthesis pathway. Mice lacking FXR exhibit bile acid dyshomeostasis and metabolic disorders (35).

Other nuclear receptors may also be activated by bile acids, although higher concentrations are often required, indicating that they may be more relevant under pathological conditions. PXR is a promiscuous transcription factor important in the metabolism of xenobiotics. It is stimulated by pharmacological reagents, environmental toxicants, bacterial metabolites and the secondary bile acid, LCA. Consistent with a role under pathological conditions, activation of PXR downregulates bacteria which metabolize bile acids and thereby modify bile acid homeostasis (36). In addition to 1,25-dihydroxy vitamin D_3_ and certain dietary ligands, LCA acts as endogenous ligands for VDR (37), a classic nuclear receptor that mediates several biological functions mainly related to calcium homeostasis and bone maintenance. Similar to FXR, ligand-induced activation of VDR facilitates interaction with retinoid X receptor and DNA binding (37). VDR is highly expressed throughout the digestive tract, where it has been found to induce expression of CYP3A and the multidrug resistance-associated protein-3 (37, 38), two enzymes known to metabolize toxic LCA, preventing its re-uptake and ensuring excretion in the feces (39).

Bile acids also bind to membrane expressed G-Protein-Coupled Bile Acid Receptor 1 (GPBAR1), also called Takeda G-protein-coupled receptor 5 (TGR5) (40). TGR5 is a member of the G-protein-coupled receptor family, which promotes cyclic adenosine monophosphate (cAMP) synthesis by adenylate cyclase upon activation. This subsequently activates the protein kinase-A pathway, thereby inducing the expression of its target genes (40). LCA is the most potent natural agonist of TGR5, which is expressed at high levels in the liver and intestinal tissue (40), but is also found in many other tissue types. Both conjugated and unconjugated bile acids bind to TGR5, with secondary bile acids, LCA and DCA being most potent (40). Functionally, TGR5 activation is associated with glucose metabolism, neuronal function, immune system control and liver regeneration (41). Other membrane receptors such as sphingosine 1-phosphate receptor (S1PR2) (42) and fibronectin receptor (α5β1 integrin) (43) are also activated by bile acids to stimulate intracellular signaling. Cytosolic ileal lipid binding proteins bind to intracellular bile acids, shuttling them to heteromeric OSTα-OSTβ transporters, which efficiently export them to the portal circulation. To a lesser extent, multidrug resistance-associated protein-3 basolaterally exports native and modified (glucuronidated or sulphated) bile acids from the enterocyte (44).

## Irritable Bowel Syndrome

IBS is a clinically diverse disorder, with a multifactorial etiology. Global prevalence varies from ~1% to more than 45%, with between 5-10% reported for Europe, the United States and China (45). IBS is the most widespread gastrointestinal disorder in the western world (46). Characterized by chronic, recurrent visceral pain and discomfort, individuals with IBS can be categorized according to predominant bowel habits. Subtypes include IBS with constipation (IBS-C), IBS with diarrhea (IBS-D), mixed or alternating IBS and unsubtyped phenotypes (46). In addition to genetic, epigenetic (47), immunological (48), gender differences (49) and food hypersensitivity (50, 51) being reported in individuals with IBS, an increased prevalence of adverse life events and comorbid mood disorders such as anxiety, depression and somatoform disorders (52, 53) are also common. It is generally accepted that dysfunction of the bi-directional gut-brain signaling axis contributes to symptom manifestation (54, 55). Indeed, chronic activation of the hypothalamic-pituitary-adrenal (HPA) stress axis has been identified in individuals with IBS (52, 56).

In a number of clinical studies, bile acid dyshomeostasis has been detected in individuals with IBS (summarized in **Table 1**). It has consistently been reported that individuals with diarrhea-predominant IBS have elevated concentrations of fecal primary bile acids (57–60, 62, 63). Changes in bile acid profiles were also linked with IBS-D symptoms, such as the defecation frequency (57, 58, 61, 62) and abdominal pain (60, 62, 63). The link with IBS-C is not as strong, although fecal LCA was decreased in this subset of individuals (58). Deconjugated bile acids can drive phylum level shifts, increasing firmicutes and decreasing bacteroidetes (64). Individuals with IBS have an altered microbiome, where the ratio of fecal firmicutes to bacteroidetes is increased (65) and changes in the gut microbiome (66, 67) are important contributors to the pathophysiology of IBS. Several studies have examined microbial profiles and bile acid pools in IBS and found that bacteria with functions in bile acid transformation were modified in IBS-D patients (57). Others detected distinct changes in bacterial profiles. In IBS-D, *E. coli* (60) and clostridia-rich microbiota were elevated and associated with excessive bile acid secretion (61), whereas the abundance of *ruminococcaceae* was decreased (62).

**Table 1:** Clinical studies investigating bile acid levels and microbial profiles in IBS.

	Bile acid profiles	IBS Symptoms	Microbial profiles
Duboc et al. (57)	Levels of fecal primary bile acids were elevated in IBS-D patients (n=14).	Primary bile acid levels were correlated with stool consistency and frequency.	Changes in bacterial profiles were detected in IBS-D. Some of the changes related to bacteria with a role in bile acid transformation.
Shin et al. (58)	Levels of fecal unconjugated primary bile acids were elevated in IBS-D (n=31). Fecal LCA was elevated in IBS-C patients (n=30)	Total levels of unconjugated bile acids were correlated to IBS phenotype (stool number and form). The correlation was stronger in IBS-D as compared to IBS-C.	Not investigated.
Camilleri et al. (59)	Subgroups of IBS-D patients (n=64) were identified with increased or normal levels of total fecal bile acids.	IBS-D patients with increased levels of bile acids presented with more pathophysiological changes such as fecal fat and changes in intestinal permeability.	Not investigated.
Dior et al. (60)	Circulating primary bile acids were elevated in both IBS-D (n=16) and IBS-C (n=15) patients. Fecal primary bile acids were elevated in IBS-D.	Abdominal pain was correlated with serum and fecal primary bile acid concentrations.	Escherichia coli was increased in IBS-D. Bacteroides and Bifidobacterium were increased in IBS-C patients.
Zhao et al. (61)	24.5% of IBS-D patients (n=290) exhibited excessive excretion of total fecal bile acids.	Total fecal bile acid levels were correlated with increased defecation frequency and decreased stool consistency.	Clostridia-rich microbiota was linked to excessive bile acid excretion in IBS-D.
Wei et al. (62)	Primary bile acids were increased, and secondary bile acids were decreased in IBS-D patients (n=55).	Defecation frequency was associated with primary bile acid concentrations. Visceral pain sensitivity was negatively correlated with CDCA.	The abundance of *Ruminococcaceae* was decreased in IBS-D patients. The changes were negatively correlated with primary and positively correlated with secondary bile acids.
Wei et al. (63)	Fecal primary bile acids were increased in IBS-D (pilot study). Mucosal expression of TGR5 was increased in IBS-D.	Fecal primary bile acids were correlated with severity of diarrhea. IBS-D patients with higher expression of TGR5 had more severe and more frequent abdominal pain.	Not investigated.

The above table summarizes key findings relating to circulating and fecal bile acid levels in individuals with irritable bowel syndrome (IBS). Associations with IBS symptomology and, if investigated, changes in microbial profiles in the gut lumen are listed.

## Bidirectional Gut-Brain Signaling Axis

In health, specialized innate and adaptive immune mechanisms are important in priming the gut against possible attack from luminal pathogens. An intact epithelial barrier and primed immune response excludes both commensal and non-commensal bacteria, restricting them to the external environment of the gut lumen, although it has been reported that the presence of bacterial products in the lamina propria is actually important for maintaining homeostasis in the enteric nervous system (68). However, interoceptive signaling relating to the luminal environment of the intestines are reported to the central nervous system (CNS) (69), thus, an intrinsic cross-barrier signaling mechanism would enable signaling between luminal factors and host physiological systems.

### Gut-Brain Axis

Bidirectional gut-to-brain signaling involves the peripheral nervous system, endocrine and immune mediators (70–72). Sympathetic and parasympathetic efferent nerves synapse with neurons in the neural plexi of the enteric nervous system that innervate both the submucosal and muscle layers (73, 74), thereby influencing intestinal secreto-motor activity. Two afferent neuronal subtypes underpin sensory function within the gut. The first, extrinsic primary afferents, have somata that are external to the gut and signal to the CNS. The second subtype, intrinsic primary afferent neurons (IPANs) have somata that are embedded within the gut wall and are primary afferents for secretory and motility reflexes. Both respond to changes in luminal content (75, 76) and evidence exists to support the presence of functional synapses between myenteric soma and vagal afferents, with the implication that they are the first neural link in the microbiota-gut-brain signaling axis (77).

Vagal afferents are believed to transmit information about the luminal environment through the sensitivity of its sensory endings to microbial metabolites (78). Indeed, vagal signaling has been implicated in altered central expression of neurotransmitters and changed behaviors evoked by ingestion of putative probiotics (79, 80). Behavioral changes in germ-free mice, which are born and raised under sterile conditions, implicate the critical role of microbes in the normal development of immune, endocrine and neural physiology (81). Moreover, IPANs are less excitable in germ-free mice (82), intimating microbes use neurally-mediated gut-to-brain pathways. Mechanosensory spinal afferents terminate in the serosa, muscularis and mucosa (83) and many visceral afferents are polymodal, sensing more than one stimulus modality (84). We and others have recorded changes in the excitability of vagal afferents in the jejunum (80, 85) and colon (6, 86, 87) following exposure to bacterial strains or their secretory products. These afferents are appropriately positioned to sense chemo-nociceptive signals (88) such as luminal bile acids. Indeed, TGR5 has been detected on IPANs (89) and has also been implicated in gut-to-brain satiety-related signaling *via* the vagus nerve (90). Germ-free mice exhibit increased levels of bile acids and increased activation of TGR5 (91).

### Enteroendocrine Cells

Epithelial stem cells give rise to four distinct cellular lineages, including specialized chemosensory enteroendocrine cells. Embedded amongst other enterocytes in the epithelial layer, enteroendocrine cells sense the presence of nutrients and other stimulatory factors in the luminal contents. Although they represent only ~1% of the epithelial cell population, collectively these cells make up the largest hormone-secreting organ in the body. These polarized cells have an apical side which faces the gut lumen and a hormone-secreting side that releases endocrine factors basolaterally when activated (92). There are more than twenty different enteroendocrine cell types in the gut.

Serotonin (5-HT)-secreting enterochromaffin cells are one such chemosensory cell type and they are coupled to sensory nerves. Catecholamines and microbially-produced short-chain fatty acids, such as butyrate and isobutyrate (93) can stimulate 5-HT release. 5-HT has a profound impact on bowel function by influencing neural modulation of intestinal smooth muscle *via* 5-HT_3_ and 5-HT_4_ receptors. Expression of SERT, proteins responsible for the reuptake of 5-HT following synaptic transmission into mucosal enterocytes and presynaptic neurons, is decreased in IBS, an aspect that may have genetic origins (94), and contributes to the pathophysiology of IBS (95). Abnormalities in postprandial serotonin release have been linked to IBS subtype, with impaired postprandial serotonin release detected in IBS-C patients, while increased plasma serotonin was identified in individuals with IBS-D (96, 97).

Recent research has detected expression of TGR5 in enterochromaffin cells in the colon but not the small intestine (98). In mice, 5-HT stimulated an increase in bile excretion but concomitantly increased ASBT expression, leading to lower levels of colonic bile acids (99), which is linked to decreased colonic motility, increased water reabsorption and constipation.

Glucagon-like peptide-1 (GLP-1) and peptide YY (PYY)-secreting L-cells are electrically excitable biosensors integrated into the epithelium. They express a plethora of receptors including receptors for GABA (100), short-chain fatty acids (101) and also 5-HT (98), for which agonists can be derived from luminal bacteria. Moreover, L-cells in rodents (102, 103) and humans (104, 105) express both FXR and TGR5, making them promising candidates for sensing, translating and transmitting signals from the colonic lumen to the mammalian nervous system (106, 107). Indeed, in isolated human enterocytes, 73% of GLP-1 expressing enteroendocrine cells expressed TGR5, whereas only 16% of GLP-1 negative cells expressed this receptor, suggesting that L-cells are the predominant cellular transducers of TGR5-mediated bile acid-mediated signals. Inhibition of ileal ABSTs using elobixibat resulted in elevated levels of circulating GLP-1, likely through increased interaction of colonic bile acids with GLP-1 secreting L-cells (108). Exposure to LCA and other bile acid TGR5 agonists resulted in increased cAMP, calcium rises and secretion of GLP-1 from L-cells (109). Interestingly, TGR5 is expressed on the basolateral membrane of L-cells (102, 105, 110), indicating that bile acids must be transported across the epithelium to stimulate GLP-1 release from L-cells. Bile acids also activate nuclear FXR in enterocytes (111). It appears that conjugated bile acids stimulate the TGR5/GLP-1 pathway, whereas CDCA activates the FXR/FGF19 pathway, decreasing expression of GLP-1 (105), emphasizing the differential receptor binding affinities for bile acids. This evidence is consistent with the existence of an epithelial-neural pathway, which could facilitate signaling from bile acids in the gut lumen to the host neurophysiological system *via* cellular transducers in the epithelium with precise, temporal transmission of sensory signals (106).

### Immune Cells

Bile acids are noted for their antimicrobial properties which prevents small intestinal bacterial overgrowth (23). This may be mediated through direct cytotoxicity (21) or by stimulating innate immune mechanisms (22). Furthermore, diarrhea resulting from exposure to high levels of bile acids in the colon may be part of the innate immune response to protect the intestinal epithelium from cytotoxic bile acids, such as LCA (24). TGR5, FXR and VDR expression has been detected in innate immune cells such as monocytes, macrophages, dendritic cells and natural killer cells (29–31). Indeed, TGR5 activation in monocytes and macrophages evoke a reduction in the release of pro-inflammatory cytokines and phagocytic activity (112–114). Bile acids appear to have an important role in fine-tuning the immune response to the divergence of antigens that the gut is exposed to. Generally, the responses tend to be inhibitory and favor gut tolerance (115). In addition to altered microbial profiles, indicators of immune activation, such as elevated levels of proinflammatory cytokines and increased infiltration of immune cells to the lamina propria, have been described as part of the pathology of IBS (55, 116). Microbiota-induced changes in colonic bile acid pools could subsequently modify TGR5 or FXR function in immune cells, although the details of this potential mechanism are yet to be explored.

### Brain-Gut Signaling

An adaptive or allostatic response to a perceived environmental threat underpins the stress response and is initiated by release of corticotropin-releasing factor from the hypothalamus to stimulate HPA activity. Chronic activation of the HPA axis has, however, been associated with altered bowel morphology, function and visceral pain sensitivity (117, 118) and is frequently co-morbid in individuals with IBS (119). Moreover, stress and activation of the HPA axis are linked to microbial dysbiosis (120), demonstrating the two-way communication between the brain and the gut. Although circulating bile acids don’t normally cross the blood brain barrier, when serum bile acids are increased, as is the case in cholestasis, they can gain access to the central nervous system through a leaky blood brain barrier and become concentrated in the hypothalamus (121). An animal model of cholestasis demonstrated the transport of specific bile acids into hypothalamic neurons resulting in decreased expression and secretion of corticotropin-releasing factor with overall suppressive effects on HPA activity that is mediated through glucocorticoid receptors (122). Others have shown that supraphysiologic concentrations of bile acids in the periphery suppress hepatic glucocorticoid clearance and, in this way, inhibit activity of the HPA axis (123). Thus, bile acids may modify central regulation of gut function and thereby contribute to the pathophysiology of IBS.

## Bile Acids in the Manifestation of IBS Symptoms

Colonic exposure to excess bile acids, which may be due to loss of bile acid transporters in the ileum causing bile acid malabsorption (124), overproduction of bile acids, or as a secondary consequence of gastrointestinal disease, has been linked to increased intestinal secretion and motility. Bile acid malabsorption typically results in chronic watery diarrhea, a symptom also characteristic of IBS-D, although the selenium-homocholic acid taurine test (Se-HCAT) test, which detects increased colonic bile acid exposure can differentiate between the two disorders (125). Fecal bile acids are raised in ~25% of individuals with IBS-D (126) resulting in accelerated colonic transit, which is linked with diarrhea and visceral pain sensitivity. Moreover, colestipol treatment, which binds bile acids and prevents reabsorption in the ileum, improved IBS symptoms (127).

### Altered Bowel Function: Absorpto-Secretory Function

Modification of epithelial permeability and stimulation of pro-secretory pathways involving cAMP is likely to underlie the manifestation of bile acid-evoked watery diarrhea (128, 129), while bile acid subtype and conjugation status will determine bile acid specific effects (130, 131). The capacity of specific bile acids to increase epithelial permeability may be neurally regulated (132), however, if tight junctions are compromised, conjugated bile salts, which, generally do not act as secretagogues (28, 133), can gain access to the epithelial basolateral membrane and subsequently evoke an increase in cytosolic calcium leading to chloride-mediated secretion (134). Increased excretion and synthesis of serum C4 (7α-hydroxy-4-cholesten-3-one), which stimulates bile acid synthesis is thought to underpin this observation (135). Changes in bile acid secretion, induced by cholecystectomy increase the risk of developing functional bowel disorders such as IBS (136), with a prevalence of IBS-D (137). As bile acids accelerate colonic transit, it is unsurprising that lower concentrations of bile acids in the colon correlated with decreased gut transit time (138). Delivery of bile acids to the colon is decreased in patients with cholestasis and this has been linked to the manifestation of constipation (139), although the link between bile acids and IBS-C is less clear than in IBS-D patients (140). Nonetheless, a reduction in the concentration of fecal bile acids was detected in a subset (15%) of individuals with IBS-C when compared with healthy volunteers. Moreover, the IBS group had notable decreases in DCA and CDCA but increased LCA. The potential for the therapeutic use of orally administered bile acids in chronic constipation (141) and IBS-C (142) or by inhibiting the active uptake of bile acids in the ileum using an ASBT inhibitor, such as elobixibat (143, 144) is being explored.

### Intestinal Motor Function

Although the modulatory effects of bile acids on absorpto-secretory function are most obvious, both primary and secondary bile acids can also modify intestinal motor function. In a patient study, modest increases in stool bile acids were noted as an underlying factor in the onset of diarrhea in individuals diagnosed with IBS-D, but who did not have bile acid malabsorption, and it was found that this was mediated by an increase in gut motility (145). Perfusion studies assessing the effects of the secondary bile acid, DCA, on feline colonic motility showed that it had significant excitatory effects on contractility (146). A similar study in humans showed that DCA caused a considerable increase in the contractile force of the colon when it was compared to the effects of the known muscarinic acetylcholine receptor agonist, carbachol. CA and CDCA had negligible effects (147). Moreover, in rabbit colonic tissue, the inhibitory actions of voltage gated sodium channel blockers implicated cholinergic and alpha adrenergic intramural neurons in the pro-kinetic actions of DCA (148). Studies in mice showed that TGR5, is highly expressed in the myenteric plexus, which regulates intestinal motility. When TGR5 was knocked-out, bile acids did not stimulate longitudinal muscle contractility, whole-gut transit was slower and fecal water content was reduced. The investigators deduced that the prokinetic effects of bile acids in the colon are mediated by TGR5 expressed on 5-HT-secreting enterochromaffin cells and calcitonin gene-related peptide-secreting intrinsic primary afferent neurons (89).

### Visceral Hypersensitivity

In addition to altered bowel habit, individuals with IBS often present with bloating and abdominal pain indicative of visceral hypersensitivity. Recently reported findings from a rodent study have implicated Nerve Growth Factor (NGF) and transient receptor potential vanilloid channel (TRPV1) nociceptors in a signaling pathway where bile acids could modulate visceral pain signals. NGF, an important mediator in the generation and maintenance of pain, was upregulated following exposure to bile acids and this was mediated through activation of FXR. As mast cells synthesize, store and secrete NGF, the authors suggested that bile stimulated NGF release from mast cells, which, in turn, activated nociceptors and induced visceral hypersensitivity (149). TRPV1-expressing sensory fibers are increased in individuals with IBS (150) and visceral pain sensitivity has been linked to activation of mucosal mast cells in proximity to colonic sensory nerves in a similar cohort (151), providing a signaling axis by which bile acids could modulate visceral pain perception. In healthy volunteers, introduction of DCA (152) and CDCA (153) to the colon increased visceral pain sensitivity to rectal distension, however, thus far no correlation between fecal bile acids and abdominal pain severity and frequency in IBS-D patients, has been detected (62).

## Discussion

**Figure 2:**
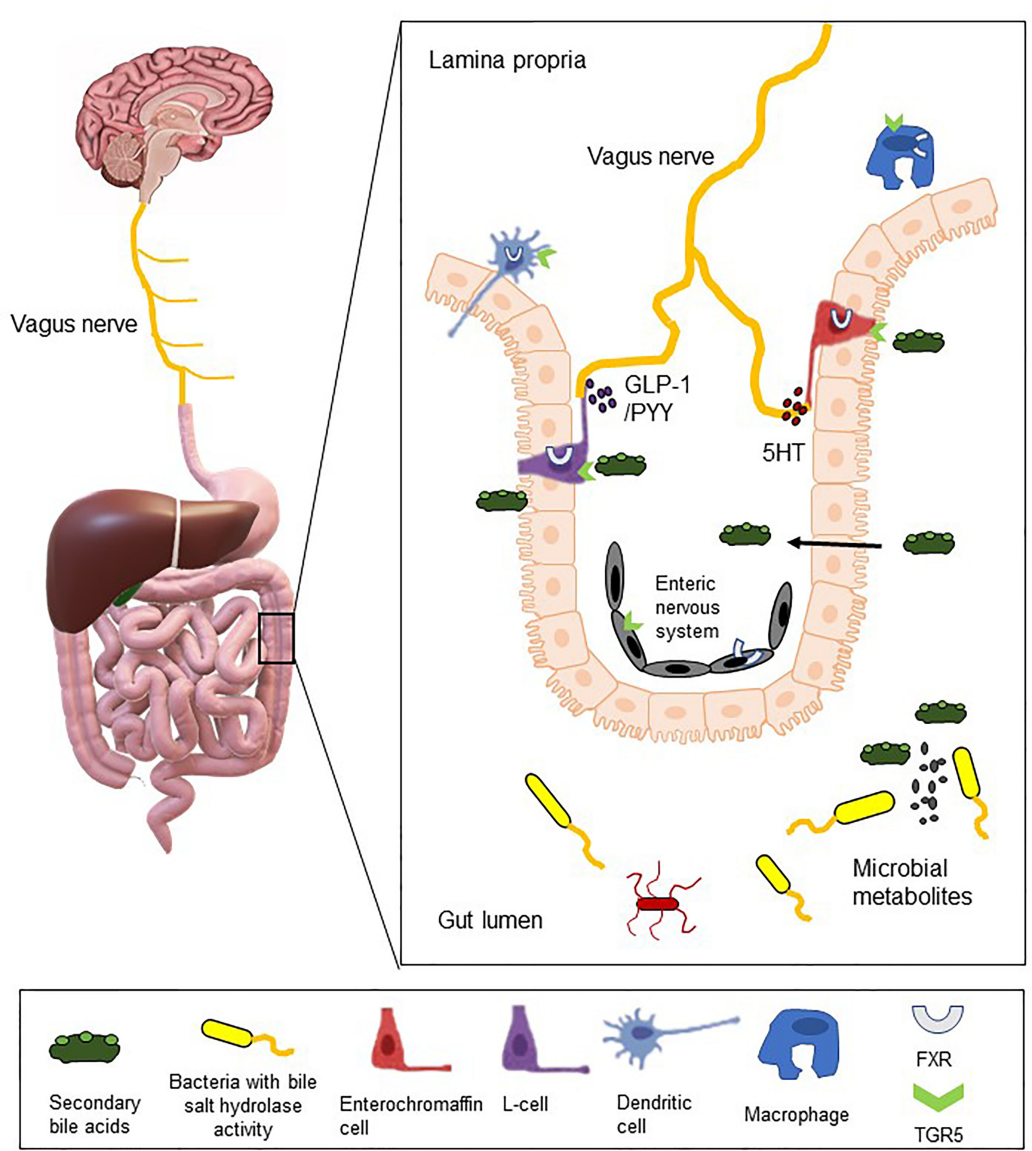
Bile acids as bioactive molecules in the gut-brain signaling axis. The illustration depicts interactions between colonic microbes with bile salt hydrolase activity and luminal bile acids. These bile acids may subsequently bind to bile acid receptors (FXR and TGR5 illustrated), which are expressed on 5-HT-secreting enterochromaffin cells, GLP-1-secreting L-cells, immune cells and on intrinsic and extrinsic neural cells. Through direct or indirect mechanisms, bile acids may act as endocrine factors or neuromodulatory agents and thereby modify local gut function and/or gut-to-brain signaling.

Research into the etiology of IBS recognizes the complexity of this multifactorial and heterogenous bowel disorder. There is growing evidence to support a role for bile acids in the pathophysiology of IBS, through interactions with the microbiome and host sensory and/or immune cells or through direct actions on the peripheral and central nervous system (**Figure 2**). The perfunctory actions of bile acids as detergents in the small intestine belie the complex functions instilled in these bioactive signaling molecules. The bidirectional relationship with gut microbiota impacts on microbial profiles and on the bile acid pool itself. The striking pro-secretory and prokinetic actions of bile acids on colonic function are consistent with the manifestation of IBS-D symptoms, whereas reduction in exposure to bile acids, though less researched, is more consistent with decreased gut secretion and transit, as presented in individuals with IBS-C. Elevated levels of excreted primary bile acids have consistently been detected in individuals with IBS-D and a growing number of studies have linked changes in fecal and serum bile acids with IBS symptomology, and changes in bacterial profiles with specific links to bacteria with bile acid transformation functionality (**Table 1**).

Mechanistically, bile acid receptors are expressed on intrinsic and extrinsic nerves, which could facilitate direct neurally-mediated changes in gut function. Moreover, when bile acids are at supraphysiological levels, they can breach the blood brain barrier to modify the HPA axis, which in turn, can modulate intestinal function and luminal microbes. However, bile acid receptors are also detected on enteroendocrine and immune cells, which could both act as signal transducing intermediaries, secreting factors which subsequently modify colonic activity either through direct actions or by modulating neural regulation of the gut. Further complexity lies in the variability of responses evoked by conjugated or unconjugated, primary or secondary bile acids. As bile acids have emerged as effectors in microbe-host signaling and can directly and indirectly modulate gut homeostasis, these bioactive molecules should not be overlooked as the pathophysiology of IBS is elucidated. Indeed, interventions to modify colonic exposure to bile acids could reveal effective therapeutic options for this functional bowel disorder.

## Author Contributions

RD, QX, and DO’M prepared this review together. All authors contributed to the article and approved the submitted version.

## Funding

DO’M is a funded investigator in APC Microbiome Ireland, which is supported by Science Foundation Ireland [Grant SFI/12/RC/2273]. QX is supported by CSC, PR of China.

## Conflict of Interest

The authors declare that the research was conducted in the absence of any commercial or financial relationships that could be construed as a potential conflict of interest.

The reviewer VC declared a shared affiliation with the authors to the handling editor at time of review.

## Publisher’s Note

All claims expressed in this article are solely those of the authors and do not necessarily represent those of their affiliated organizations, or those of the publisher, the editors and the reviewers. Any product that may be evaluated in this article, or claim that may be made by its manufacturer, is not guaranteed or endorsed by the publisher.
